# Clinical characteristics, outcomes and risk factors of candidial infections with critically ill patients in respiratory intensive care unit in turkey

**DOI:** 10.1186/2197-425X-3-S1-A1011

**Published:** 2015-10-01

**Authors:** İ Erayman, DM Yavşan, R Altuntaş, E Karataş, M Göktepe, T Teke, K Uzun

**Affiliations:** Necmettin Erbakan University Meram Medical Faculty, Infectious Diseaes and Clinical Microbiology, Konya, Turkey; Necmettin Erbakan University Meram Medical Faculty, Pulmonary Diseases and Critical Care Unit, Konya, Turkey

## Objectives

Candidial infection is associated with high mortality in critically ill patients. Fungal infections compose a major problem in intensive care units in both developed and developing countries. Candidemia is associated with a prolonged hospital stay, resulting in increasedcosts, and high mortality.

## Methods

This study was conducted in a 12-bed adult respiratory intensive care unit (>18 years). Clinical and laboratory data from patients with candidial infection were collected retrospectively.

## Results

Median age was 70.1 ± 14.60 years (min 18 years, max 91 years). The median APACHE II score on admission was 21.28 ± 5.39. A total of 405 episodes were identified from 2007 to 2014. The rates of candidial infections were urinary tract infection (67.6%), blood stream infection (23.5%) and pneumonia (8.9%). The fungi identified were yeast (54.32%), *C. albicans* (26.40%), *C. tropicalis* (5.91%), *C. glabrata* (6.91%), *C. parapsilosis* (3.20%), *C. krusei* (1.23%), Candida kefyr (0.98%) and other Candida species (1.23%) (Table1). Candida albicans accounted for 57.29% of all *Candida* species. Fluconazole resistance was found in 35.42%. The susceptibility to amphotericine B was 96.94%. Itraconazole resistance was 70.22%. None of all patiens was using previously antifungal therapy. Primary therapy included monotherapy with fluconazole (n = 99), caspofungin (n = 14), anidulafungin (n = 8) and voriconazole (n = 8). Combination therapy was infrequently used (n = 6). The mortality rate was 77.6% (n = 184). The use of broad-spectrum antibiotics was 85.2%. The presence of an intravascular device was 70% (n = 164). The use of parenteral (n = 66), enteral nutrition (n = 46) and both (n = 98) were 27.8%, 19.4% and 41.4% respectively. The median mechanic ventilation days were 13.3 ± 15.96 days. The median RICU length of stay (LOS) was 24.8 ± 24.19 days. The most patients (61.18%) came to RICU from other departments and care units.

## Conclusions

In conclusion, candidial infections associated with high mortality, APACHE II score, LOS, ventilation days and use of broad-spectrum antibiotics.

**Table 1:** Candida Species in Culture Types.

n(%)	UTI (n:274)	BSI (n:95)	BAL (n:36)	Total
Yeast	201 (73.36)	13 (13.68)	6 (16.67)	220 (54.32)
C.albicans	39 (14.23)	50 (52.63)	17 (47.22)	106 (26.17)
C. tropicalis	7 (2.55)	14 (14.74)	3 (8.33)	24 (5.93)
C. glabrata	13 (4.74)	8 (8.42)	7 (19.44)	28 (6.91)
C. parapsilosis	1 (0.36)	9 (9.47)	3 (8.33)	13 (3.21)
C. krusei	4 (1.46)	1 (1.05)	0	5 (1.23)
C. kefyr	4 (1.46)	0	0	4 (0.99)
Other C. species	5 (1.82)	0	0	5 (1.23)
Total	274 (67.6)	95 (23.5)	36 (8.9)	405

